# A Repeated-Measures Study: Does Acute Exposure to Simulated Moderate Altitude Alter Wingate Performance and Skeletal Muscle Oxygenation Differently in Sprinters and Middle- and Long-Distance Runners?

**DOI:** 10.7759/cureus.115683

**Published:** 2026-09-02

**Authors:** Tambi Medabala, Ankalayya Bobbara, Vishavdeep Kaur

**Affiliations:** 1 Department of Physiology, Sports Authority of India, Netaji Subhas National Institute of Sports (NSNIS), Patiala, IND; 2 Department of Physiology, Maharishi Markandeshwar Institute of Medical Sciences and Research, Ambala, IND

**Keywords:** acute hypoxia, anaerobic performance, muscle oxygenation, near-infrared spectroscopy, simulated altitude, sub-elite athletes, wingate test

## Abstract

Background: Acute hypoxia may preserve initial explosive power while impairing power maintenance. This study examined graded acute simulated altitude effects on Wingate performance and vastus lateralis oxygenation in sprinters and middle- and long-distance (MD/LD) athletes.

Methods: Forty-four Indian sub-elite athletes, comprising 22 sprinters and 22 MD/LD athletes, completed a 30-second Wingate test under normoxia and simulated altitudes of 2000 m and 3000 m. At least 72 hours separated the trials. Muscle oxygenation was assessed using near-infrared spectroscopy. Data were analyzed using 2×3 mixed-design repeated-measures analysis of variance, Bonferroni-adjusted comparisons, and Spearman correlations.

Results: At 3000 m, peak power decreased by 4.87-5.03%, average power by 7.24-9.84%, and minimum power by 11.78-15.72%. Power drop increased by 14.47-15.63%. Altitude significantly affected all Wingate outcomes (all p<0.001). The effects were largest for power drop (ηp²=0.972), minimum power (ηp²=0.954), and average power (ηp²=0.913), showing greater impairment of power maintenance. Group×altitude interactions were significant for average power (p<0.001), minimum power (p<0.001), and power drop (p=0.023), but not peak power (p=0.980). Oxygenated hemoglobin-myoglobin signal (O₂Hb), total hemoglobin (tHb), and tissue saturation index (TSI) decreased, whereas deoxygenated hemoglobin-myoglobin (HHb) increased (all altitude effects p<0.001; ηp²=0.950-0.998). At 3000 m, TSI was lower in sprinters than in MD/LD athletes (39.72±7.83% vs. 46.17±7.57%; adjusted p=0.025). Average power loss correlated with TSI change (ρ=-0.645; adjusted p<0.001) and HHb change (ρ=-0.707; adjusted p<0.001).

Conclusion: Acute simulated altitude impaired power maintenance and fatigue resistance more strongly than initial explosive power. Changes in TSI and HHb were associated with average power loss, particularly at 3000 m. These correlations do not establish causality. Sprinters and MD/LD athletes showed different response patterns, but neither group demonstrated a consistent overall advantage. Average power, minimum power, power drop, and muscle oxygenation may be considered together when evaluating anaerobic performance under hypoxic conditions.

## Introduction

Exposure to acute hypoxia reduces inspired oxygen pressure and arterial oxygen saturation. This restricts systemic and muscular oxygen availability and increases cardiorespiratory and metabolic strain [1-3]. Its effect on performance depends on the hypoxic dose and task duration. Exercise capacity generally declines, but efforts lasting less than two minutes appear comparatively resistant [4]. Supramaximal output may therefore remain preserved despite marked arterial desaturation and muscle deoxygenation [5,6].

The 30-second Wingate test characterizes several dimensions of anaerobic performance. Peak power represents the highest early output; average power reflects power maintenance; minimum power captures late-test output; and power drop quantifies fatigue-related decline. Peak and mean power were preserved under moderate hypoxia in sprinters, kayakers, and triathletes [5-7]. Conversely, sprint work and force declined during greater or graded hypoxic exposure [8,9]. Peak power alone may therefore conceal a progressive failure to sustain output. Average power, minimum power, and power drop may reveal the broader functional effect of hypoxia.

Near-infrared spectroscopy (NIRS) portable devices provide continuous and non-invasive assessment of local muscle oxygenation. The oxygenated hemoglobin-myoglobin signal (O₂Hb) reflects oxygen delivery relative to utilization, whereas deoxygenated hemoglobin-myoglobin (HHb) is associated with oxygen extraction. Total hemoglobin (tHb) indicates changes in local blood volume, while the tissue saturation index (TSI) represents relative tissue oxygen saturation. Hypoxia commonly produces a larger decline in O₂Hb, greater HHb accumulation, and lower muscle oxygen saturation during exercise [3,5,10,11]. NIRS can therefore identify peripheral oxygen stress even when external power is preserved. However, it cannot completely separate hemoglobin from myoglobin signals [10,12].

Most existing studies have examined one athletic group, untrained controls, small mixed samples, or a single hypoxic condition. Sprinters were compared with untrained men only at approximately 2,000 m, whereas graded-altitude research used a small and heterogeneous athletic sample [5,8]. Wingate evidence among triathletes also comes from one simulated altitude [7]. Direct comparisons between sprinters and middle- and long-distance (MD/LD) athletes across graded simulated altitudes remain limited. Combining Wingate and NIRS measurements may clarify whether training background modifies fatigue and peripheral oxygen responses.

Based on the above milieu, this study intended, as the primary objective, to examine the effects of acute simulated altitude exposure at 2,000 m and 3,000 m, compared with normoxia, on Wingate performance and NIRS-derived vastus lateralis muscle oxygenation in sprinters and MD/LD athletes and, as the secondary objective, to determine whether altitude-induced changes in muscle oxygenation are associated with declines in anaerobic performance.

## Materials and methods

This repeated-measures experimental study intended to examine the acute effects of simulated altitude on anaerobic performance and skeletal muscle oxygenation in male athletes. All 44 participants completed the Wingate anaerobic test under each of the three experimental conditions. The conditions were administered in a fixed ascending order: normoxia, simulated 2,000 m, and simulated 3,000 m. No experimental trials were missing. A minimum interval of 72 hours was maintained between consecutive trials. The study was conducted in the Department of Exercise Physiology, Sports Authority of India, Netaji Subhas National Institute of Sports (NSNIS), Patiala, India (study period: March 2025 to June 2025). One day before the first experimental trial, each participant completed one familiarization session. The session included orientation to the hypoxic chamber environment and its safety procedures, adjustment of the cycle ergometer, the five-minute warm-up, application of resistance equivalent to 7.5% of body mass, standardized test instructions, and NIRS device placement. Each participant also completed a full 30-second Wingate test using the cycle ergometer located inside the chamber. The familiarization session was separate from the 60-minute pre-exercise exposures conducted before the experimental hypoxic trials. Data collection was conducted at NSNIS, Patiala, after obtaining prior administrative approval from the competent institutional authority, while ethical oversight was provided by the Institutional Ethics Committee of Maharishi Markandeshwar Institute of Medical Sciences and Research (approval number: IEC-3145), where the study was undertaken as part of the first author's PhD research.

Participants and sample size calculation

The final sample included 44 Indian male sub-elite athletes. Participants were classified as sprinters (n=22) or MD/LD athletes (n=22). Eligible athletes were 20-30 years of age. They had participated at least at the national level and had a minimum sport-specific training history of four to five years. All athletes were actively engaged in regular training during the study period.

Athletes were excluded if they had sustained a musculoskeletal injury during the preceding six months. Those with cardiovascular or respiratory complaints were also excluded. Medical history and physical examination were used to assess eligibility. Height and body mass were recorded before testing using standardized measuring equipment (SECA, model 286, SECA, GmbH & Co. KG., Hamburg, Germany).

An a priori sample size calculation was performed using G*Power Version 3.1.9.4 (Heinrich Heine University Düsseldorf, Düsseldorf, Germany). The F-test family and repeated-measures analysis of variance (ANOVA) with a within-between interaction procedure were selected, with the group×altitude interaction specified as the target effect. The calculation assumed a moderate effect size of Cohen's f=0.25, an α level of 0.05, a statistical power of 0.80, two athlete groups, three repeated measurements, a correlation of 0.50 among repeated measurements, and a nonsphericity correction of ε=0.60. The estimated minimum total sample was 40 participants, with an actual power of approximately 0.814. An additional 10% was included to account for possible attrition or incomplete trials, increasing the recruitment target to 44 participants. All 44 participants completed the three experimental conditions, resulting in a final analytical sample of 22 MD/LD athletes and 22 sprinters.

Simulated altitude exposure

Hypoxic exposure was produced using a normobaric hypoxic chamber manufactured by L.O.T. LOWOXYGEN Technology GmbH (Berlin, Germany). The chamber had an approximate volume of 90 m³. Three inspired oxygen conditions were examined: normoxia at a fraction of inspired oxygen (FiO₂) of 20.9% (0.209), simulated 2000 m at an FiO₂ of 16.4% (0.164), and simulated 3000 m at an FiO₂ of 14.5% (0.145). Before each experimental trial, participants were instructed to refrain from exercise and caffeine-containing products for 24 hours. They were asked to maintain their habitual diet and usual hydration practices and to obtain at least six hours of sleep during the preceding night. All experimental trials were conducted between 10:00 and 13:00. The same pre-test instructions and testing-time window were applied across the three experimental conditions.

Before each experimental trial, including normoxia, participants remained inside the same chamber for 60 minutes under the assigned oxygen condition. The conditions were FiO₂ 20.9% for normoxia, FiO₂ 16.4% for simulated 2,000 m, and FiO₂ 14.5% for simulated 3,000 m. Chamber temperature was maintained at approximately 16°C, with relative humidity between 40% and 50%. FiO₂ was continuously monitored throughout the pre-exposure period and Wingate test using the chamber's integrated oxygen sensor and control system. The Wingate test was performed inside the chamber while the assigned oxygen condition was maintained. Barometric pressure was not recorded.

Wingate anaerobic test

The Wingate test was performed using a Monark 894E cycle ergometer which was kept inside the hypoxic chamber (Monark Exercise AB, Vansbro, Sweden). Before testing, each athlete completed a five-minute cycling warm-up. The participant then performed a 30-second all-out cycling effort. Resistance was set at 7.5% of individual body mass. Participants were instructed to pedal maximally throughout the test.

Performance data were obtained from the ergometer software. Peak power was defined as the highest power output recorded during the test. Average power represented the mean power maintained across the complete 30-second effort. Minimum power was the lowest power recorded during the test. Power drop represented the relative decline from peak power to minimum power and was calculated as follows: $\text{Power Drop (\%)}=[(\text{Peak Power}-\text{Minimum Power})/\text{Peak Power}]\times100$. Peak power, average power, and minimum power were normalized to body mass and expressed as watts per kilogram (W/kg). Power drop was expressed as a percentage.

NIRS measurement of muscle oxygenation

Muscle oxygenation was measured continuously using a wireless PortaMon NIRS device (Artinis Medical Systems, Elst, Gelderland, Netherlands) positioned over the vastus lateralis. The sensor was secured firmly to reduce movement and ambient light interference. A stable resting signal was recorded before each test. Recording then continued throughout the 30-second Wingate effort under all three oxygen conditions. Data were acquired and exported using OxySoft software (Artinis Medical Systems, Elst, Gelderland, Netherlands). O₂Hb, HHb, tHb, and TSI were obtained for analysis. Changes in O₂Hb, HHb, and tHb were calculated as the during-Wingate value minus the pre-Wingate baseline. TSI was expressed as a percentage (data acquired as per the standard procedures of the Original Equipment Manufacturer (OEM) Manual). Negative O₂Hb values indicated reduced muscle oxygenation, while positive HHb values indicated greater muscle deoxygenation.

Statistical analysis

IBM SPSS Statistics for Windows, Version 25.0 (IBM Corp., Armonk, New York, United States) was employed for results analysis. Continuous data were summarized as mean±standard deviation. Variables that did not meet the normality assumption were presented as median and interquartile range. Normality was assessed using the Shapiro-Wilk test. Homogeneity of variance was examined using Levene's test. Baseline group differences were assessed using the independent-samples t-test or Mann-Whitney U test, as appropriate.

A 2×3 mixed-design repeated-measures ANOVA was performed separately for each Wingate and NIRS variable. Athlete group was the between-subject factor and included sprinters and MD/LD athletes. Altitude was the within-subject factor and included normoxia, 2000 m, and 3000 m. The analysis examined the main effects of group and altitude and the group×altitude interaction. Partial eta squared (ηp²) was reported as the measure of effect size. Significant effects were examined using Bonferroni-adjusted pairwise comparisons. When an interaction was significant, follow-up comparisons assessed whether the magnitude of altitude-related change differed between the athlete groups.

Spearman's rank-order correlation was used to examine associations between physiological change scores and the deterioration in Wingate performance. Average power loss was calculated as the normoxic value minus the altitude value. Power drop, HHb, and TSI changes were calculated as altitude minus normoxia. Bonferroni adjustment was applied across the 20 correlation tests.

Two simultaneous multiple linear regression models were constructed to predict the percentage decline in average power from normoxia to 2000 m and from normoxia to 3000 m. Percentage decline was calculated as follows: $\text{Average Power decline (\%)}=[(\text{Normoxic Average Power}-\text{Altitude Average Power})/\text{Normoxic Average Power}]\times100$. The predictors were athlete group, TSI decrease (calculated as normoxia minus altitude), and HHb increase (calculated as altitude minus normoxia). Athlete group was coded as MD/LD=0 and sprinter=1. All predictors were entered simultaneously. Model performance was assessed using R², adjusted R², and the overall F-test. Variance inflation factors were examined for multicollinearity. All statistical tests were two-tailed. Statistical significance was accepted at p<0.05.

## Results

The analysis included 44 male athletes, with 22 MD/LD athletes and 22 sprinters. Age was similar between the groups (p=0.267). MD/LD athletes were taller than sprinters (median: 168 cm vs. 164 cm; p<0.001) and had greater body mass (77.14±6.93 kg vs. 71.37±9.12 kg; p=0.023). Participant characteristics are summarized in Table 1.

**Table 1:** Participant characteristics and baseline group comparisons Data are presented as mean±standard deviation, except height, which is median (interquartile range). p<0.05 was considered statistically significant.

Variable	Middle- and long-distance athletes (n=22)	Sprinters (n=22)	Test	Statistic	P-value
Sex, male, n (%)	22 (100%)	22 (100%)	Not applicable	-	-
Age (years)	25.36±2.56	24.50±2.54	Independent-samples t-test	t=1.12	0.267
Height (cm)	168 (167-170)	164 (161-166)	Mann-Whitney U test	U=85.00	<0.001
Body mass (kg)	77.14±6.93	71.37±9.12	Independent-samples t-test	t=2.36	0.023

Wingate performance and repeated-measures analysis

Peak power showed modest reductions from normoxia to 3000 m in MD/LD athletes and sprinters (5.03% and 4.87%, respectively). Average power declined by 9.84% in MD/LD athletes and 7.24% in sprinters. The largest descriptive reduction occurred in minimum power (15.72% and 11.78%). Power drop increased progressively and was 15.63% higher in MD/LD athletes and 14.47% higher in sprinters at 3000 m than in normoxia. Thus, late-test power and fatigue-related measures changed more than initial peak power.

Simulated altitude had a significant main effect on every Wingate outcome (all p<0.001). The largest effects were observed for power drop (ηp²=0.972), minimum power (ηp²=0.954), and average power (ηp²=0.913). Each pairwise altitude comparison was significant for every outcome (all adjusted p<0.001). No overall group effect was significant. The group×altitude interaction was not significant for peak power (p=0.980), but it was significant for average power (p<0.001), minimum power (p<0.001), and power drop (p=0.023). Interaction follow-up tests showed group differences in the magnitude of average power change from normoxia to 3000 m (p<0.001) and from 2000 m to 3000 m (p=0.003). All three minimum power contrasts were significant (p=0.001, p=0.001, and p=0.009). The corresponding power drop contrasts were not significant after adjustment (Tables 2-3).

**Table 2:** Wingate performance across simulated altitude conditions Values are presented as mean±standard deviation. The analysis included 22 middle- and long-distance athletes and 22 sprinters.

Variable	Athlete group	Normoxia	2000 m	3000 m
Peak power (W/kg)	Middle- and long-distance athletes (n=22)	11.13±1.47	10.89±1.47	10.57±1.56
	Sprinters (n=22)	11.71±1.60	11.49±1.35	11.14±1.33
Average power (W/kg)	Middle- and long-distance athletes (n=22)	8.03±0.54	7.78±0.50	7.24±0.44
	Sprinters (n=22)	8.15±0.54	7.97±0.53	7.56±0.50
Minimum power (W/kg)	Middle- and long-distance athletes (n=22)	4.77±0.65	4.50±0.60	4.02±0.55
	Sprinters (n=22)	5.01±0.60	4.81±0.58	4.42±0.54
Power drop (%)	Middle- and long-distance athletes (n=22)	57.01±6.99	59.67±6.55	65.92±6.32
	Sprinters (n=22)	55.90±13.26	58.48±13.30	63.99±13.14

**Table 3:** Repeated-measures ANOVA and interaction follow-up comparisons for Wingate performance A 2×3 mixed-design repeated-measures ANOVA was performed. Athlete group was the between-subject factor, comprising middle- and long-distance athletes (n=22) and sprinters (n=22). Altitude was the within-subject factor and included normoxia, 2000 m, and 3000 m. All overall pairwise comparisons between altitude conditions were significant after Bonferroni adjustment (all adjusted p<0.001). ᵃInteraction follow-up p-values are presented in the following order: normoxia to 2000 m, normoxia to 3000 m, and 2000 to 3000 m. These comparisons test whether the magnitude of altitude-related change differed between the athlete groups. Follow-up comparisons were not performed for peak power because the group×altitude interaction was not significant. p<0.05 was considered statistically significant.

Variable	Group effect, p (ηp²)	Altitude effect, p (ηp²)	Group×altitude, p (ηp²)	Interaction follow-up, adjusted pᵃ
Peak power	0.186 (0.041)	<0.001 (0.399)	0.980 (0.000)	Not performed
Average power	0.174 (0.044)	<0.001 (0.913)	<0.001 (0.184)	0.458; <0.001; 0.003
Minimum power	0.076 (0.073)	<0.001 (0.954)	<0.001 (0.238)	0.001; 0.001; 0.009
Power drop	0.657 (0.005)	<0.001 (0.972)	0.023 (0.086)	1.000; 0.105; 0.058

NIRS muscle oxygenation across simulated altitude

During the Wingate test, O₂Hb, tHb, and TSI decreased progressively with altitude, whereas HHb increased. The main effect of altitude was significant for all NIRS outcomes (all p<0.001; ηp²=0.950-0.998). TSI was the only outcome with a significant overall group effect (p=0.021; ηp²=0.120). Significant group×altitude interactions were found for O₂Hb (p<0.001), HHb (p<0.001), and TSI (p=0.002), but not for tHb (p=0.746). Direct group comparisons were non-significant except for TSI at 3000 m, where sprinters had a lower value than MD/LD athletes (39.72±7.83% vs. 46.17±7.57%; adjusted p=0.025). All pairwise altitude comparisons were significant for each NIRS outcome (all adjusted p<0.001) (Tables 4-5).

**Table 4:** NIRS-derived muscle oxygenation and repeated-measures analysis across simulated altitude (descriptive NIRS values) Values are presented as mean±standard deviation. Δ indicates change from the pre-Wingate value to the during-Wingate value. Negative O₂Hb and tHb values indicate a reduction, while positive HHb values indicate an increase. NIRS: near-infrared spectroscopy; HHb: deoxygenated hemoglobin-myoglobin; O₂Hb: oxygenated hemoglobin-myoglobin signal; tHb: total hemoglobin; TSI: tissue saturation index

Variable	Group	Normoxia	2000 m	3000 m
O₂Hb ΔµM	Middle- and long-distance athletes (n=22)	-4.03±3.36	-7.46±3.50	-11.42±3.59
O₂Hb ΔµM	Sprinters (n=22)	-6.43±7.95	-10.19±7.99	-14.49±8.05
HHb ΔµM	Middle- and long-distance athletes (n=22)	3.10±2.76	6.00±2.75	9.38±2.81
HHb ΔµM	Sprinters (n=22)	5.06±6.61	8.29±6.63	12.20±6.68
tHb ΔµM	Middle- and long-distance athletes (n=22)	-0.93±3.47	-1.46±3.55	-2.04±3.59
tHb ΔµM	Sprinters (n=22)	-1.36±6.20	-1.90±6.21	-2.44±6.21
TSI (%)	Middle- and long-distance athletes (n=22)	59.68±8.19	52.71±7.67	46.17±7.57
TSI (%)	Sprinters (n=22)	54.55±8.19	47.45±7.42	39.72±7.83

**Table 5:** NIRS-derived muscle oxygenation and repeated-measures analysis across simulated altitude: repeated-measures ANOVA and adjusted follow-up comparisons The table reports the results of the 2×3 mixed-design repeated-measures ANOVA and Bonferroni-adjusted follow-up comparisons. ᵃDirect group p-values are presented in the following order: normoxia, 2000 m, and 3000 m. ᵇGroup change p-values are presented in the following order: normoxia to 2000 m, normoxia to 3000 m, and 2000 to 3000 m. These comparisons test whether the magnitude of altitude-related change differed between the athlete groups. All overall pairwise comparisons between altitude conditions were significant (all adjusted p<0.001). Statistical significance was accepted at p<0.05. NIRS: near-infrared spectroscopy; HHb: deoxygenated hemoglobin-myoglobin; O₂Hb: oxygenated hemoglobin-myoglobin signal; tHb: total hemoglobin; TSI: tissue saturation index

Variable	Group p (ηp²)	Altitude p (ηp²)	Group×altitude p (ηp²)	Direct group pᵃ	Group change pᵇ
O₂Hb	0.149 (0.049)	<0.001 (0.998)	<0.001 (0.484)	0.611; 0.460; 0.338	<0.001; <0.001; <0.001
HHb	0.131 (0.053)	<0.001 (0.993)	<0.001 (0.353)	0.625; 0.437; 0.236	<0.001; <0.001; 0.005
tHb	0.783 (0.002)	<0.001 (0.950)	0.746 (0.007)	1.000; 1.000; 1.000	1.000; 1.000; 0.848
TSI	0.021 (0.120)	<0.001 (0.984)	0.002 (0.137)	0.132; 0.077; 0.025	1.000; 0.007; 0.002

NIRS-performance change-score correlations

For the normoxia to 2000 m contrast, power drop increase correlated inversely with the change in HHb (ρ=-0.549; adjusted p=0.002). Average power loss also correlated inversely with signed TSI change (ρ=-0.468; adjusted p=0.027) and HHb change (ρ=-0.480; adjusted p=0.020). The associations were generally stronger for the normoxia to 3000 m contrast. The strongest association was between average power loss and HHb change (ρ=-0.707; adjusted p<0.001), followed by signed TSI change (ρ=-0.645; adjusted p<0.001). Only correlations that remained significant after correction are reported (Table 6).

**Table 6:** NIRS-performance change-score correlations Values are presented as Spearman rank correlation coefficients (n=44). Average power loss was calculated as normoxia minus altitude. Power drop, HHb, and TSI changes were calculated as altitude minus normoxia. Negative TSI values therefore indicate lower tissue saturation at altitude. Adjusted p-values used Bonferroni correction across 20 comparisons. Only associations that remained significant after adjustment are shown. NIRS: near-infrared spectroscopy; HHb: deoxygenated hemoglobin-myoglobin; O₂Hb: oxygenated hemoglobin-myoglobin signal; tHb: total hemoglobin; TSI: tissue saturation index

Altitude contrast	Performance variable	Physiological variable	ρ	Adjusted p
Normoxia to 2000 m	Power drop increase (%)	HHb change during Wingate (µM)	-0.549	0.002
Normoxia to 2000 m	Average power loss (W/kg)	TSI change during Wingate (percentage points)	-0.468	0.027
Normoxia to 2000 m	Average power loss (W/kg)	HHb change during Wingate (µM)	-0.480	0.020
Normoxia to 3000 m	Power drop increase (%)	HHb change during Wingate (µM)	-0.476	0.022
Normoxia to 3000 m	Average power loss (W/kg)	TSI change during Wingate (percentage points)	-0.645	<0.001
Normoxia to 3000 m	Average power loss (W/kg)	HHb change during Wingate (µM)	-0.707	<0.001

Regression predicting average power decline

The normoxia to 2000 m regression model explained 15.7% of the variance in average power decline but was not statistically significant (adjusted R²=0.094; F(3,40)=2.485; p=0.075). Although TSI decrease had a significant coefficient in this model (B=-0.456; β=-0.321; p=0.033), it should be interpreted cautiously because the overall model was not significant. The normoxia to 3000 m model explained 68% of the variance and was statistically significant (adjusted R²=0.656; F(3,40)=28.392; p<0.001). Athlete group (B=-1.374; β=-0.317; p=0.015) and TSI decrease (B=-0.975; β=-0.646; p<0.001) were significant independent predictors at 3000 m. Variance inflation factors ranged from 1.01 to 2.54 (Tables 7-8).

**Table 7:** Multiple regression predicting percentage decline in average power: model summary and ANOVA Simultaneous multiple linear regression was used to predict the percentage decline in average power. The table presents the model summary and omnibus ANOVA results. The analysis included 44 athletes. Statistical significance was accepted at p<0.05. R²: coefficient of determination; SEE: standard error of estimate

Contrast	R²	Adjusted R²	SEE	F-statistic	Model p
Normoxia to 2000 m	0.157	0.094	2.048	F(3,40)=2.485	0.075
Normoxia to 3000 m	0.680	0.656	1.285	F(3,40)=28.392	<0.001

**Table 8:** Multiple regression predicting percentage decline in average power: regression coefficients Simultaneous multiple linear regression was used to predict the percentage decline in average power. The three predictors were athlete group, TSI decrease, and HHb increase. Athlete group was coded as middle- and long-distance athletes=0 and sprinters=1. TSI decrease was calculated as normoxia minus altitude, whereas HHb increase was calculated as altitude minus normoxia. The 95% CI are reported for the unstandardized regression coefficients. The analysis included 44 athletes. Statistical significance was accepted at p<0.05. B: unstandardized coefficient; β: standardized coefficient; HHb: deoxygenated hemoglobin-myoglobin; TSI: tissue saturation index; VIF: variance inflation factor; -: not applicable

Contrast	Predictor	B	95% CI for B	β	t	p	VIF
2000 m	Constant	10.980	-3.374 to 25.334	-	1.546	0.130	-
2000 m	Group (sprinters=1)	-0.357	-2.345 to 1.631	-0.084	-0.363	0.718	2.54
2000 m	TSI decrease (percentage points)	-0.456	-0.874 to -0.038	-0.321	-2.203	0.033	1.01
2000 m	HHb increase (µM)	-1.618	-6.371 to 3.135	-0.159	-0.688	0.495	2.53
3000 m	Constant	22.293	16.043 to 28.543	-	7.209	<0.001	-
3000 m	Group (sprinters=1)	-1.374	-2.464 to -0.284	-0.317	-2.547	0.015	1.94
3000 m	TSI decrease (percentage points)	-0.975	-1.283 to -0.667	-0.646	-6.401	<0.001	1.27
3000 m	HHb increase (µM)	0.099	-0.686 to 0.884	0.030	0.255	0.800	1.73

## Discussion

This study examined acute normobaric hypoxia, Wingate performance, and vastus lateralis oxygenation in sprinters and MD/LD athletes. All Wingate outcomes deteriorated progressively from normoxia to 2000 m and 3000 m. At 3000 m, peak power decreased by 4.87-5.03%, whereas average power fell by 7.24-9.84% and minimum power by 11.78-15.72%. Power drop increased by 14.47-15.63%. Thus, hypoxia affected late-test output more strongly than initial explosive power. O₂Hb, tHb, and TSI decreased, while HHb increased. NIRS changes were also associated with performance deterioration, particularly at 3000 m.

The graded performance decline supports an altitude-dose response. Brief exercise is generally less sensitive to acute hypoxia than longer work [4]. Peak or mean power was preserved during hypoxic Wingate and sprint protocols in sprinters, kayakers, and triathletes [5-7]. Early power was relatively resistant, but power maintenance was not. Similar impairment in sprint work or force has been reported during graded simulated altitude and longer sprint tasks [8,9]. Comparable selectivity has been observed in resistance exercise, where maximal strength remained stable but muscular endurance declined at 3000 m or above [13]. As the Wingate effort continues, oxidative ATP contribution increases, phosphocreatine availability declines, and fatigue-related metabolites accumulate. Reduced oxygen availability may therefore become more important during the later seconds. Peak power alone can underestimate the functional effect of hypoxia.

The NIRS responses accompanied the observed performance pattern and provided complementary evidence of altered local muscle oxygenation under simulated altitude. From normoxia to 3000 m, the magnitude of the O₂Hb reduction increased by 183.4% in MD/LD athletes and 125.3% in sprinters (altitude p<0.001; group×altitude p<0.001). TSI decreased by 22.6% and 27.2%, respectively (altitude p<0.001; group×altitude p=0.002). These changes indicate a worsening imbalance between local oxygen availability and muscular demand. HHb increased by 202.6% in MD/LD athletes and 141.1% in sprinters (altitude p<0.001; group×altitude p<0.001). This finding is compatible with greater muscle deoxygenation. The magnitude of the tHb reduction increased by 119.4% and 79.4%, respectively (altitude p<0.001; group×altitude p=0.746). However, the absolute tHb reduction was relatively small. It may indicate an additional alteration in local blood volume or perfusion. Comparable responses have been reported during hypoxic Wingate exercise, repeated upper limb sprints, intermittent handgrip exercise, and cycling [3,5,10,11]. In several studies, muscle oxygenation worsened despite preserved external performance. NIRS variables had very large altitude effects, whereas peak power was comparatively resistant. NIRS may therefore detect peripheral oxygen stress that is not evident from maximal power alone.

No Wingate outcome showed a significant overall group effect, and peak power showed no group×altitude interaction. However, average power, minimum power, and power drop showed significant interactions. MD/LD athletes had larger descriptive reductions in average and minimum power at 3000 m. Sprinters had a 1.902-percentage-point smaller adjusted average power decline, yet their TSI was lower at 3000 m. At 3000 m, the exploratory regression indicated a smaller adjusted average power decline in sprinters, whereas TSI was lower in sprinters than in MD/LD athletes. This divergent pattern may suggest a training group difference in the relationship between local muscle oxygenation and power maintenance. However, the present study did not directly measure oxygen extraction, metabolic utilization, or physiological mechanisms responsible for tolerance to deoxygenation. Therefore, this interpretation remains hypothesis-generating. Greater muscle deoxygenation alongside preserved Wingate power has also been reported in sprint-trained athletes [5]. Nevertheless, most direct group comparisons for O₂Hb and HHb were not significant, and tHb showed no interaction. These findings represent different response patterns rather than a uniform advantage for either group. Training status may modify hypoxic physiology, but individual variability remains important [14,15].

A larger fall in TSI was associated with greater average power loss. This relationship was stronger at 3000 m (ρ=-0.645) than at 2000 m (ρ=-0.468). Because TSI change was calculated as altitude minus normoxia, a more negative value represented greater desaturation. Larger HHb increases were associated with smaller performance losses. The strongest relationship was observed between HHb change and average power loss at 3000 m (ρ=-0.707). Greater HHb may reflect a compensatory capacity to extract or utilize available oxygen. This is consistent with greater deoxygenation occurring alongside maintained sprint output in earlier studies [5,11]. However, HHb is not a direct measure of extraction alone. The regression models explained 15.7% and 68% of average power decline at 2000 m and 3000 m, respectively, and athlete group and TSI change were independent predictors, whereas HHb was not. TSI may represent the net oxygen delivery mismatch more effectively. These associations do not establish causation.

The findings have practical relevance for acute altitude exposure. Peak power should not be used alone to assess readiness for high-intensity work. Average power, minimum power, and power drop are more sensitive to fatigue development. Training density should be reduced initially, particularly near 3000 m. Adequate recovery may help preserve sprint quality. Repeated Wingate performance was maintained with seven-minute recovery under moderate hypoxia, whereas shorter-recovery sprint work deteriorated at 3000 m [7,8].

The study has important strengths. Its within-participant design enabled direct comparison of three oxygen conditions. Both athlete groups were equally represented. Wingate and NIRS measurements were obtained during the same trials. Multiple performance indices, effect sizes, adjusted comparisons, corrected correlations, and regression models strengthened the analysis.

Several limitations remain. Acute normobaric exposure may not reproduce natural altitude, acclimatization, or competition. The findings may not generalize beyond Indian sub-elite athletes aged 20-30 years. Baseline height and body mass differed between groups, although power was normalized to body mass. The cycling Wingate test may not represent every sport. NIRS was recorded from one superficial muscle site. It cannot measure whole-limb oxygenation, directly quantify blood flow, or fully separate hemoglobin from myoglobin [10,12]. The experimental conditions were conducted in a fixed ascending order and were not randomized or counterbalanced. Consequently, simulated altitude exposure was not fully separable from the testing sequence, and potential order, learning, or residual fatigue effects cannot be excluded. Although participants underwent familiarization and at least 72 hours separated consecutive trials, these procedures could not eliminate possible sequence-related effects.

## Conclusions

Acute hypoxia exposure impaired power maintenance and fatigue resistance more strongly than initial explosive power among the athletes. Physiologically, lower O₂Hb, tHb, and TSI, together with higher HHb, indicated reduced local oxygen availability and greater muscle deoxygenation. Changes in muscle oxygenation were associated with average power loss. However, the correlational analyses do not establish that altered muscle oxygenation caused the decline in performance. Sprinters and MD/LD athletes showed different response patterns, but neither group demonstrated a consistent overall advantage. Therefore, peak power alone may underestimate the functional impact of acute altitude exposure. Average power, minimum power, power drop, and muscle oxygenation may be considered together when evaluating anaerobic performance under hypoxic conditions.
